# Genetic Predisposition to Hepatocarcinogenesis in Inbred and Outbred Mouse Lines Selected for High or Low Inflammatory Response

**DOI:** 10.1155/2019/5298792

**Published:** 2019-03-31

**Authors:** Lilian Rego de Carvalho, Andrea Borrego, José Ricardo Jensen, Wafa Hanna Koury Cabrera, Aline Marques Santos, Orlando Garcia Ribeiro, Nancy Starobinas, Marcelo De Franco, Tommaso A. Dragani, Giacomo Manenti, Olga Célia Martinez Ibañez

**Affiliations:** ^1^Laboratory of Immunogenetics, Instituto Butantan, São Paulo, Brazil; ^2^Department of Pathology, University of Brasilia, Brasilia, Brazil; ^3^Diagnostic Section, Pasteur Institute, São Paulo, Brazil; ^4^Department of Predictive and Preventive Medicine, Fondazione IRCCS Istituto Nazionale dei Tumori, Milan, Italy

## Abstract

AIRmax and AIRmin mouse strains phenotypically selected for high and low acute inflammatory responsiveness (AIR) are, respectively, susceptible or resistant to developing hepatocellular carcinoma (HCC) induced by the chemical carcinogens urethane and diethylnitrosamine (DEN). Early production of TNF-*α*, IL-1*β*, and IL-6 in the liver after DEN treatment correlated with tumor development in AIRmax mice. Transcriptome analysis of livers from untreated AIRmax and AIRmin mice showed specific gene expression profiles in each line, which might play a role in their differential susceptibility to HCC. Linkage analysis with SNP markers in F2 (AIRmax×AIRmin) intercross mice revealed two quantitative trait loci (QTL) in chromosomes 2 and 9, which are significantly associated with the number and progression of urethane-induced liver tumors. An independent linkage analysis with an intercross population from A/J and C57BL/6J inbred mice mapped regions in chromosomes 1 and 7 associated with the progression of urethane-induced liver tumors, evidencing the heterogeneity of HCC genetic control.

## 1. Introduction

Many human cancers such as those affecting the liver are etiologically related to processes of inflammation and/or chronic infection. Human hepatocellular carcinoma (HCC) is the third most common cause of cancer mortality and the seventh in terms of cancer incidence worldwide [1]. The incidence of HCC varies widely according to racial and ethnic groups and to geographic location, as a result of the influence of genetic factors and of regional variations in exposure to risk factors [2]. Among the most important risk factors of liver cancer are hepatitis B, chronic hepatitis C virus infection, hereditary hemochromatosis, and cirrhosis of almost any cause. Tobacco and alcohol abuse, environmental toxins and dietary factors, diabetes mellitus, nonalcoholic fatty liver disease, alpha-1 antitrypsin deficiency, and autoimmune hepatitis are all associated with increased risk for HCC [3]. The incidence of HCC is approximately three times higher in men than in women, and most of the experimental models confirm this sex difference [4].

We developed two mouse lines that differ widely in inflammatory responsiveness to investigate the genetic control of inflammatory processes. These lines were produced by bidirectional phenotypic selection on the basis of the intensity of the local acute inflammatory response (AIR) to a nonimmunogenic inflammatory stimulus (Bio-Gel P-100 polyacrylamide beads) and were called AIRmax (for high response) and AIRmin (for low response) [5]. The acute inflammatory response was measured by the number of infiltrated cells and protein concentration in the 24-hour inflammatory exudate induced by subcutaneous Bio-Gel injection. The foundation population was constituted by mixing 8 inbred mouse laboratory lines, and after about 25 generations of selective breeding, the phenotypic divergence between AIRmax and AIRmin lines reached 20 to 30 times for the number of infiltrating leukocytes (85% neutrophils) and 2.5-fold for exudate protein concentration [6].

At the end of the selection process, these mouse lines also differed in susceptibility to several chemically induced tumors. AIRmax are more susceptible than AIRmin to colon carcinogenesis [7] while AIRmin are more susceptible to skin, lung, and kidney carcinogenesis [8–10]. The two strains have distinct gene expression profiles in the bone marrow and in the normal lung [11, 12] which correlated with their differential inflammation capacity and tumor susceptibility, respectively.

To better investigate the genetic relationship between inflammation and cancer susceptibility, we carried out linkage analyses using a large pedigree of about 700 F2 (AIRmax×AIRmin) intercross mice. In this experiment, newborn mice were treated with urethane, the inflammatory reaction to subcutaneous Bio-Gel injection was measured 3 months later, and tumors in internal organs were analyzed at 7 months of age. Genetic linkage analysis results of lung and kidney tumor susceptibility were published [10, 13], and here, we show the results of liver carcinogenesis.

Urethane treatment induced large and multiple liver tumors in all male AIRmax mice, whereas few AIRmin male mice developed small hepatic lesions. The liver tumor load in the whole pedigree of F2 (AIRmax×AIRmin) mice was used in the present study for linkage analysis with SNPs. Similar analysis was carried out in the fourth generation of an advanced intercross population named ABF4 between A/J and C57BL/6J inbred mice.

The transcriptome of normal livers as well as the response of AIRmax and AIRmin mice to another genotoxic chemical carcinogen, N-nitrosodiethylamine (DEN), was also analyzed in order to confirm the divergent susceptibility of these two mouse lines to liver carcinogenesis and to assess the role of tissue cells in the control of immune responses against tumors.

## 2. Methods

### 2.1. Mice and Tumor Induction

The formal stock designations of the mouse lines used are Ibut:AIRH and Ibut:AIRL at the ILAR (Institute for Laboratory Animal Research, National Academy of Sciences, USA), but they are referred to as AIRmax and AIRmin in this paper as well as in previous publications. These lines and crosses for production of the F2 (AIRmax×AIRmin) population were developed and maintained at the animal facilities of the Laboratory of Immunogenetics of the Butantan Institute (São Paulo, Brazil). Male and female 15-day-old mice were used in experiments for long-term studies of hepatic cancer development and also for analysis of cytokine production in the liver after injection of the carcinogen diethylnitrosamine (N-nitrosodiethylamine (DEN), Sigma-Aldrich, St. Louis, MO) (25 mg/kg bw ip). Urethane (carbamic acid ethyl ester, Sigma-Aldrich, St. Louis, MO) (300 mg/kg bw ip) was injected in groups of male and female mice seven days after birth. Mice were euthanized at 34 weeks after treatment for analysis of tumor formation in internal organs. Liver tumor incidence, multiplicity, and volumes (calculated by measuring the mean diameters of the lesions) were recorded. FFPE liver fragments were cut into 4 *μ*m sections and stained with hematoxylin and eosin.

We used, for linkage analysis, the liver carcinogenesis results of 693 F2 (AIRmax and AIRmin) intercross mice and of 183 F4 intercross mice (ABF4), generated by mating C57BL/6 (B) with A/J (A) mice (details in [14]). F2 (AIRmax×AIRmin) mice were treated with one dose of urethane (300 mg/kg bw ip) 7 days after birth, and ABF4 mice were treated with a single intraperitoneal injection of urethane (1 g/kg body weight) at 4 weeks of age; the mice were killed at 34 and 40 weeks after treatment, respectively.

All procedures were approved by the Institutional Animal Care and Use Committees of Butantan Institute and Istituto Tumori, and all animals received humane care, according to the criteria outlined in the “Guide for the Care and Use of Laboratory Animals” prepared by the National Academy of Sciences and published by the National Institutes of Health.

### 2.2. Cytokine Analysis

Groups of 6 AIRmax and 6 AIRmin mice were euthanized at 2, 8, and 24 h after DEN injection to analyze the acute cytokine production in the liver. Total liver homogenates were prepared by homogenizing the whole liver from each mouse in 1500 *μ*l of sterile PBS, and 100 *μ*l of supernatant from each liver homogenate was analyzed with the OptEIA Mouse ELISA Sets (BD Biosciences, San Jose, CA, USA) for IL-6, TNF-*α*, and IL-1*β* according to the manufacturer's instructions. Cytokine concentration was calculated as pg/ml for each sample.

### 2.3. Genome-Wide SNP Genotyping

Genomic DNA was extracted from tail tips using the E.Z.N.A.® Tissue DNA Kit (Omega Bio-Tek, Norcross, USA) and quantified using the Quant-iT™ PicoGreen® dsDNA Assay Kit (Invitrogen, Carlsbad, CA). SNP genotyping was carried out in all mice of both the F2 (AIRmax×AIRmin) intercross pedigree and intercrossed ABF4 mice using the 1449-SNP loci mouse medium density linkage panel (Illumina Inc., San Diego, CA) at a density of approximately three SNPs per 5 Mb across the whole genome as described in [15]. QTLs affecting the liver tumor phenotypes were mapped through genome-wide linkage analyses by interval mapping using GridQTL version 3.1.0 [16] that uses a linear model to fit phenotype data according to genotypes. Additive and dominant effects at the QTL were included with other explanatory variables such as sex and family. The significance thresholds of phenotype-genotype associations were estimated by genome-wide permutation analysis (*n* = 5000 or 10,000 permutations).

### 2.4. Affymetrix Microarray Analysis

Total RNA from the livers of untreated adult mice were isolated using the RNAspin Mini Kit (GE Healthcare, Buckinghamshire, UK). The concentrations and quality of the purified RNA were determined in the NanoDrop (Thermo Fisher Scientific) and in the Agilent 2100 Bioanalyzer (Agilent Technologies, Santa Clara, CA) equipment, respectively. The Mouse Gene 1.0 ST Array (Affymetrix, Santa Clara, CA) with 28,853 well-annotated genes was used for transcriptome analysis. The reactions of the array were performed following the manufacturers' protocols at the AFIP-UNIFESP Molecular Facility, Federal University of São Paulo, Brazil. Four biological replicates for each group were run individually. Differentially expressed genes (DEGs) were identified by unpaired one-way ANOVA within the Transcriptome Analysis Console 3.0 (TAC) software (Affymetrix) with FDR < 0.05 (false discovery rate).

### 2.5. Real-Time Quantitative RT-PCR

Microarray data were validated by quantitative real-time PCR. Amplification mixtures containing 1 *μ*l template cDNA, 12.5 *μ*l SYBR Green PCR Master Mix (Invitrogen, Carlsbad, CA, USA), and 0.3 *μ*M specific PCR primers were run on a StepOne (Applied Biosystems, Foster City, CA) machine. The mouse *Hprt* (hypoxanthine-guanine phosphoribosyltransferase) and *Ppia* (cyclophilin A) genes were used as housekeeping controls for possible differences in cDNA amounts. Target and housekeeping genes were amplified with the following primers: *Hprt* F:5′-CGTCGTGATTAGCGATGATGA-3′ and R:5′-CCAAATCCTCGGCATAATGATT-3′; *Ppia* F:5′-AGCGTTTTGGGTCCAGGAAT-3′ and R:5′-AAATGCCCGCAAGTCAAAAG-3′; *H2Ea* prime time predesigned primers set-Mm.PT.56a.42692423.g; *Defb1*: prime time predesigned primers set-Mm.PT.56a.5676756; *Vnn3* prime time predesigned primers Mm.PT.56a.9417908; *TNF-α* F:5′-TCTCATCAGTTCTATGGCCC-3′ and R:5′-GGGAGTAGACAAGGTACAAC-3′; *Il6* F:5′-GTTCTCTGGGAAATCGTGGA-3′ and R:5′-TGTACTCCAGGTAGCTATGG-3′; *Cyp1a1*: F:5′-TGGAGACCTTCCGGCATTC-3 and R:5′-GCCATTCAGACTTGTATCTCTTGTG-3′; and *Cyp1b1* F:5′TGGCTGCTCATCCTCTTTAC-3′ and R:5′-AGGTTGGGCTGGTCACTCAT-3′.

### 2.6. Statistical Analysis

Differences between means were determined by Student's *t*-test or analysis of variance (ANOVA); differences between proportions of affected and nonaffected animals were analyzed by Fisher's exact test; differences between parameters of tumor growth were analyzed by nonparametric Mann-Whitney test. Pearson correlation analysis was carried out to compare the microarray with qPCR results. *p* < 0.05 was established as the minimum level of significance.

Gene expression of each array was log-transformed to approximate Gaussian distributions and standardized over the array to adjust for systematic differences. The Significance Analysis of Microarray (SAM) software (two-class unpaired, FDR ≤ 5%) was used to detect differentially expressed genes (DEGs), in four biological replicates from each line [17].

## 3. Results

### 3.1. Urethane and DEN Treatments Induce Liver Tumors in AIRmax and AIRmin Mice

**Figure 1:**
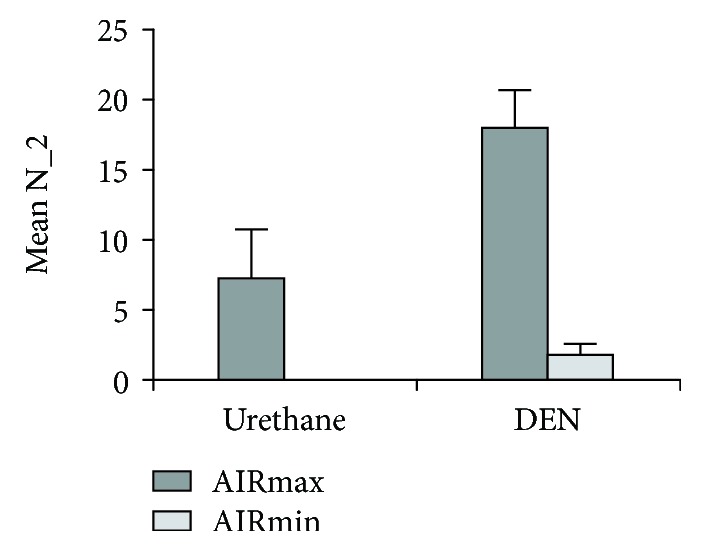
Susceptibility to liver carcinogenesis in AIRmax and AIRmin male mice. N_2 represents the number of liver tumors with diameter ≥ 2 mm. Results are expressed as mean and standard error in groups of 10 mice.

Mice were injected with urethane or DEN, and the tumor burden in internal organs was determined after 34 weeks. A reliable parameter to quantify tumor development consists in the multiplicity of lesions with a diameter ≥ 2 mm.  Figure 1 shows the mean number of ≥2 mm liver tumors after DEN or urethane injection in AIRmax and AIRmin mice. Irrespective of the carcinogen used, AIRmax mice are significantly more susceptible than AIRmin mice to developing liver tumors. Spontaneous development of liver tumors, up to the age of 1.5 years, was not observed in these mice.

**Figure 2:**
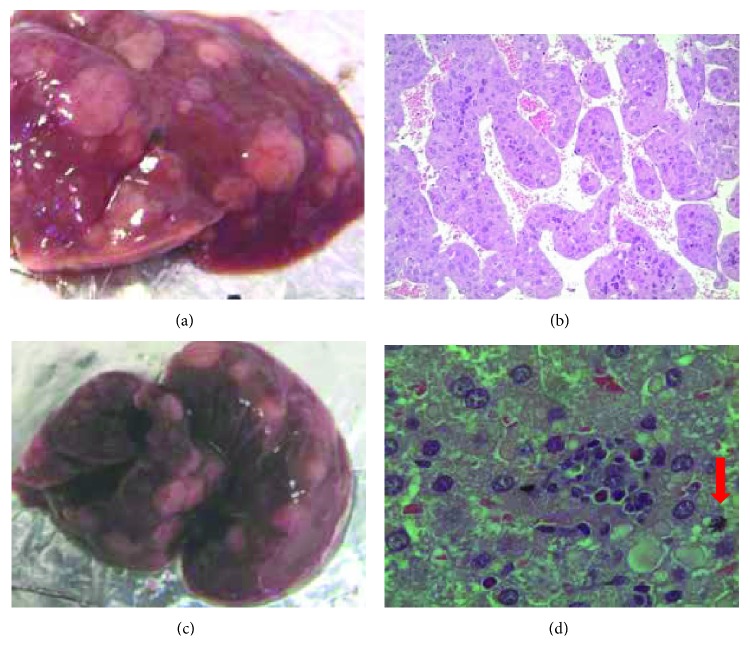
Macroscopic aspect of the AIRmax male mouse liver at 34 weeks after DEN treatment (a). HE-stained histological section showing high-grade hepatocarcinoma with atypical hepatocytes arranged in macrotrabecules forming pseudoacini (b, 200x). AIRmax liver after urethane treatment (c); HE-stained section showing high-grade hepatocarcinoma, presence of steatosis, cellular pleomorphism, alteration of lobular architecture of the hepatic parenchyma, and intratumoral inflammatory infiltrate. Arrow indicates an aberrant mitosis (d, 1000x).

Histopathological analysis revealed the occurrence of hepatocarcinomas with both treatments (Figure 2).

### 3.2. Inflammatory Cytokine Levels Increase in the Liver after DEN Treatment

**Figure 3:**
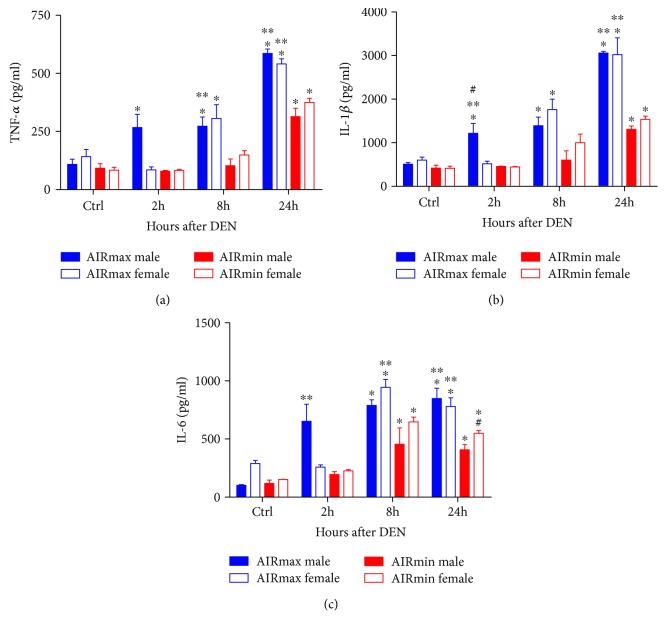
IL-6, IL-1*β*, and TNF-*α* levels in liver macerate supernatants of AIRmax and AIRmin mice treated with 25 mg/kg bw DEN (3 males and 3 females in each group). ^∗^*p* < 0.05 treated vs. control; ^&^*p* < 0.05 AIRmax vs. AIRmin; ^#^*p* < 0.05 males vs. females.

The levels of IL-6, TNF-*α*, and IL-1*β* in liver macerate supernatants were measured in order to evaluate the early DEN-induced liver inflammation. As shown in Figure 3, DEN induced cytokine production as soon as 2 h after injection. Coincidently, the cytokine levels were higher in the livers of susceptible AIRmax mice, suggesting a role for carcinogen-induced inflammatory signaling in HCC development.

### 3.3. Linkage Analysis of Hepatocarcinogenesis Modifier Loci

We aimed to map the genetic factors that contribute to the divergent susceptibility to liver carcinogenesis of AIRmax and AIRmin mice. Then, we carried out linkage analysis in an intercross F2 (AIRmax×AIRmin) population treated with urethane. Details of urethane-induced liver tumors in the groups of male mice representing the parental and F1 hybrids used for the production of the F2 intercross population are summarized in Table 1. Females were much more resistant, with only 2% incidence in the F2 population.

The logarithm of odds (LOD) thresholds for linkage of the phenotype N_2 (number of liver tumors with a diameter > 2 mm), determined by permutation analysis (*n* = 5000 permutations and sex interaction), were 4.5 and 5.4 for *α* = 0.05 and 0.01 genome-wide statistical probabilities, respectively. Because N_2 did not follow a normal distribution, the N_2 values were square root-transformed for interval mapping.

**Figure 4:**
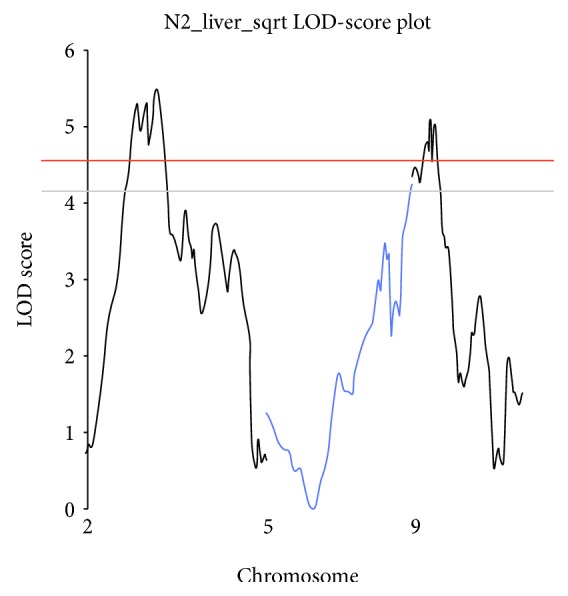
Genome-wide interval mapping of urethane-induced liver tumors. Number of liver tumors with diameter > 2 mm (N_2) revealed two significant loci at chromosomes 2 and 9 and one suggestive locus at chromosome 5; the threshold values for significant linkage at *p* < 0.05 and *p* < 0.01 are LOD scores = 4.5 and 5.4, respectively, and for suggestive linkage at *p* < 0.1 is LOD score = 4.1.

Two loci with significant LOD scores were detected in chromosomes 2 and 9. The LOD score peak = 5.5 in chromosome 2 was localized at SNP CEL_2 63143553 (70 Mb), and for the QTL in chromosome 9, the LOD score peak = 5.1 was localized at rs13480103 (17 Mb). An additional region with suggestive linkage (*p* < 0.1; LOD score = 4.2) was detected at 143 Mb in chromosome 5 (Figure 4).

**Figure 5:**
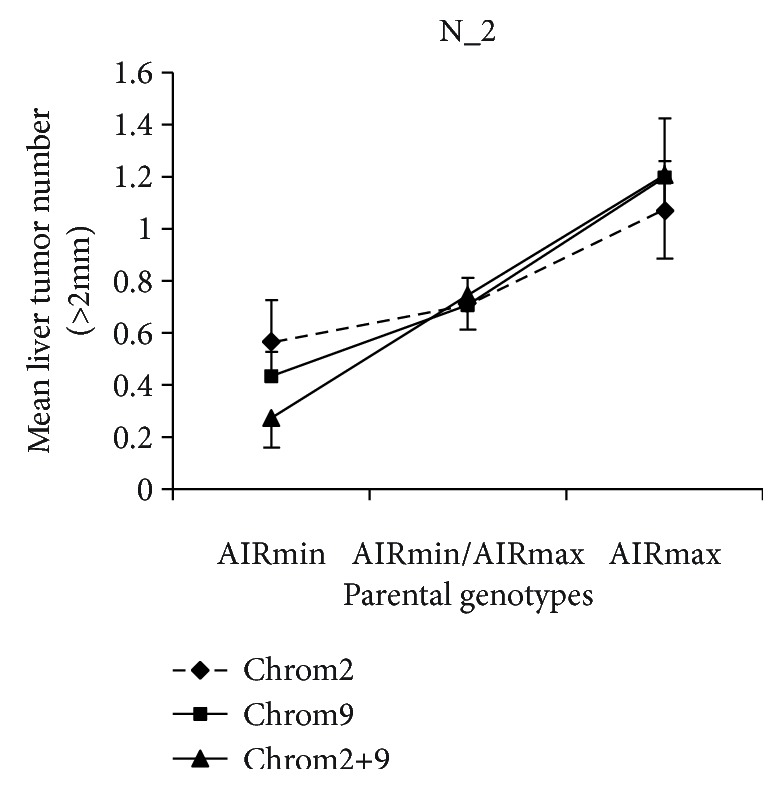
QTLs on chromosomes 2 and 9 modulate liver carcinogenesis susceptibility in F2 (AIRmax×AIRmin) mice. The graph shows the effects of the SNP genotypes located at the peak LOD score regions in chromosomes 2 and 9. Significance of differences between AIRmin- and AIRmax-associated SNP genotypes at Chrom2 ^#^*p* = 0.026, Chrom9 ^&^*p* = 0.0016, and Chrom2+9 ^∗^*p* = 0.0137.

F2 mice carrying the AIRmax-derived genotype GG (78/334) at CEL_2 63143553 in chromosome 2 had a 2-fold higher N_2 than mice carrying AIRmin-derived genotype AA (86/334; *p* = 0.026). Heterozygous mice had intermediate N_2 values. In chromosome 9, inheritance of the AA genotype (81/334) at rs13480103 from AIRmax was associated with 2.8-fold increased N_2 when compared to the group carrying the AIRmin-derived GG genotype (87/334; *p* = 0.0016). In mice carrying the combined genotypes for both SNPs (21 AIRmin-like, 87 heterozygous, and 25 AIRmax-like genotypes), the difference in N_2 values increased to 4.4-fold (*p* = 0.0137), evidencing an additive effect of the QTLs (Figure 5).

**Figure 6:**
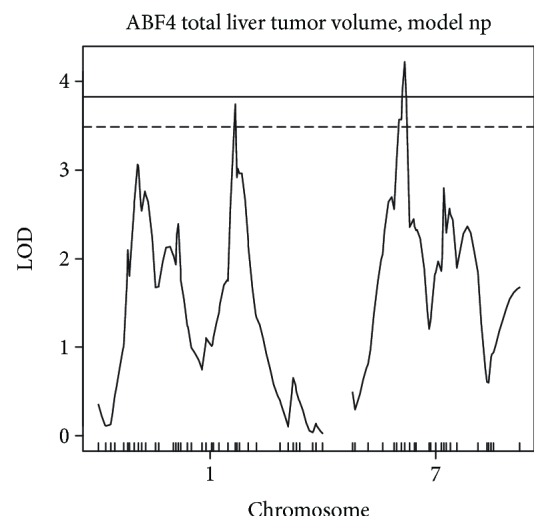
Genome-wide interval mapping of urethane-induced liver tumors; the threshold values for significant linkage at *p* < 0.05 and for suggestive linkage at *p* < 0.1 are LOD scores = 3.83 and 3.48, respectively.

We carried out a similar genetic linkage study in an advanced (A/J×C57BL/6) F4 intercross population (ABF4) with 183 urethane-treated male mice. In this population, 548 SNPs were informative (polymorphic) and nonredundant. Simple interval mapping (SIM) was performed to detect QTLs for the square root-transformed total liver tumor volume calculated by the sum of every visible liver tumor volumes. LOD thresholds for 10,000 permutations were 3.83 and 3.48 for significance at *p* < 0.05 and *p* < 0.1, respectively. SIM of urethane-induced liver tumors mapped one significant locus at 44 Mb in chromosome 7 (LOD score = 4.22) and one suggestive locus at 114 Mb in chromosome 1 (LOD score = 3.74, Figure 6). Similar results were obtained for liver tumor multiplicity.

### 3.4. Transcriptome of Normal Liver Differs in AIRmax and AIRmin Mice

**Figure 7:**
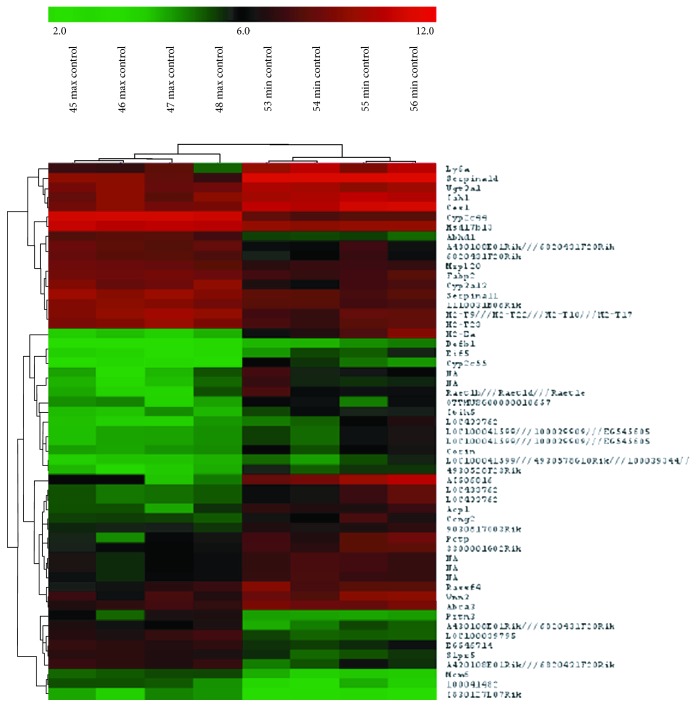
Heatmap of differentially expressed genes in normal livers from untreated AIRmax and AIRmin mice. The Mouse Gene 1.0 ST Array was used to identify sets of differentially expressed genes (*p* < 0.001). The Significance Analysis of Microarrays (SAM) using a 1.5-fold-change minimal difference revealed distinct gene expression profiles between AIRmax and AIRmin mice.

The gene expression profile of normal livers from adult untreated AIRmax and AIRmin mice, analyzed by unsupervised hierarchical clustering (considering all the 28,120 expressed probe sets), was distinct in the two mouse lines (Figure 6). We identified 145 transcripts (corresponding to 85 unique genes) discriminating the two mouse lines. Nineteen genes were upregulated and 66 were downregulated in AIRmax samples when compared to AIRmin samples (Figure 7).

Microarray results were validated by real-time PCR using the same individual mRNA samples. The fold changes between AIRmax and AIRmin for *Vnn*, *H2ea-ps*, *Defb1*, *S1pr5*, *Cyp1a1*, *Cyp1b1*, *Tnfa*, and *Il6* genes detected by real-time PCR were significantly correlated with the gene expression levels detected by microarrays (Pearson correlation *r* = 0.93).

## 4. Discussion

In the present study, we show that AIRmax male mice are highly susceptible and AIRmin mice are resistant to urethane-induced development of hepatocellular carcinoma whereas females from both strains are resistant. This phenotype was confirmed by the treatment of mice with the tobacco-related nitrosamine N-nitrosodiethylamine (NDEA or DEN), a typical genotoxic chemical carcinogen that forms DNA adducts after bioactivation by P450 isozymes, including cytochrome P450 2E1 in the liver [18, 19].

The DEN-treated animals also developed pulmonary nodules with a higher incidence in AIRmin, in agreement with the results of urethane-induced tumors [9]. Thus, the inverse susceptibility to liver versus lung carcinogenesis of AIRmax and AIRmin mice, following treatment with chemicals that induce tumors in both organs, indicates that tissue-specific genetic and environmental factors are responsible for these phenotypes [20].

In a short-term experiment, treatment with DEN induced TNF-*α*, IL-6, and IL-1*β* production in the liver. The proinflammatory cytokine IL-6, considered an acute phase protein, is increased in conditions of liver damage and hepatitis [21]. IL-6 concentrations were higher in the livers from AIRmax mice when compared to AIRmin mice. The difference was also observed for females, with variation during the time course of the experiment. Higher production of IL-1*β* and of TNF-*α* was also found in AIRmax liver macerates compared to AIRmin liver macerates, beginning 2 h after DEN injection and reaching peak concentrations at 14 and 24 h. TNF-*α* and IL-1*β* are major mediators of inflammation and display both pro- and antitumor activities. IL-1*β* has a role in tumor progression via induction of several tumor growth factors and of endothelial-mesenchymal transition [22, 23].

Several studies reported that the early DEN-elicited DNA damage contributes to necrotic liver cell death with the liberation of danger-associated molecular patterns (DAMPs). These in turn bind to toll like receptors (TLRs) that signal through the adaptor protein MyD88. MyD88 signaling induces translocation of NF-*κ*B to the nucleus and the transcription of cytokines such as IL-6 and TNF-*α* that are essential for the initiation of DEN-induced liver cancer [24–29].

It has to be pointed out that IL-6, IL-1*β*, and TNF-*α* were not detected in the liver macerates of AIRmax or AIRmin females in the first two hours after DEN injection, whereas they were present in susceptible AIRmax male mice. The liver is a sexually dimorphic organ, and gender disparity seen in HCC progression involves sex hormones. Estrogen prevents necrosis of DEN-triggered hepatocytes in female mice; at concentrations present in females but not in males, estrogen modulates or suppresses early IL-6 cytokine production, a mechanism that could account for the inhibition of liver carcinogenesis observed in females [24]. On the other side, in studies using KO mice, IL-6 antagonists or estrogen analogs to prevent HCC indicate that other mechanisms must be induced in females for liver cancer protection [25–28].

The connection between chronic inflammation and carcinogenesis has long been described for several tumor types. In normal conditions, the initial inflammatory response to injury during the acute phase is followed by an intermediate downregulation, ending in the resolution phase of inflammation. Lack of resolution is followed by a chronic phase that can be protumorigenic. In this situation, local alterations start with early activated inflammatory cells interacting with nonimmune cells (epithelial, vascular, and neuronal) via secretion of acute phase mediators. Therefore, the acute inflammation triggered in the acute phase response to injury, if unresolved, promotes tumorigenesis. In our model, the possible failure in the ability to resolve the intense carcinogen-induced acute inflammatory response in the liver of AIRmax male mice could contribute to their susceptibility to developing HCC.

Next, we studied the genetic basis for the divergent susceptibility of AIRmax and AIRmin mice to chemically induced HCC. In humans, many genetic predisposing factors to HCC are related to known environmental risk, such as obesity, alcohol use, or viral hepatitis [21, 29–31]. Nevertheless, in the absence of these factors, genetic/epidemiological studies detected an association between germline mutations in genes that underlie other diseases and increased HCC risk [32].

In mice, the complex nature of HCC genetic control was demonstrated in animal models, and several susceptible QTLs were described: *Hcs 1-8* (for HCC susceptibility locus) in chromosomes 1, 2, 5, 7, 8, 12, and 19 and *Hcf 1-2* (for HCC female susceptibility locus) in chromosomes 1 and 17. Other QTLs were associated with resistance to HCC: *Hcr2* in chromosome 10, *Hpcr3* in central chromosome 15, and *Hpcr4* in chromosome 1 [33, 34].

Here, we carried out two independent experiments for mapping urethane-induced HCC. We used 7-day-old F2 (AIRmax×AIRmin) mice and ABF4 mice in the adult phase (4 weeks after birth) aiming to compare the genetic control of urethane-induced HCC in both conditions and in different genetic backgrounds. Like many other carcinogens, urethane requires metabolic activation to become mutagenic. Compared to adults, newborn mice are more liable to the toxic and carcinogenic effects of urethane and other agents, because of lower metabolic capacity, influence of hormones, immunologic responses, and rapidly dividing cells in target tissues [35]. The different treatment protocols indeed pointed to different sets of loci in the two intercrossed populations, even if the liver tumor phenotypes used in linkage assays, scored at 34 or 40 weeks after urethane injection in F2 (AIRmax and AIRmin) and in ABF4 mice, respectively, were similar in both models.

In F2 (AIRmax×AIRmin) mice, we detected significant linkage in chromosomes 2 at 70 Mb (1-LOD score interval: 44-77 Mb) and 9 at 17 Mb (1-LOD score interval: 0-27 Mb). In the ABF4 population, one locus was detected in chromosome 7 at 52 Mb, spanning a region from 40 to 57 Mb. These three regions are novel and nonoverlapping with sets of loci reported in rat and mouse crosses. These results support the concept of genetic heterogeneity, where distinct genetic elements exerting pleiotropic effects control the same complex phenotype. Further support comes from one skin carcinogenesis study that revealed the role of genetic heterogeneity in the control of predisposition to inflammatory response and skin tumor susceptibility [36]. The association of diverse sets of loci, from different families, with the corresponding diseases is evidence that genetic heterogeneity also occurs in humans [37].

Furthermore, at these QTL regions, there are interesting coincidences with QTLs for other phenotypes described in independent experiments. In AIRmax and AIRmin mice, a linkage study including clustering by family identified a suggestive QTL for inflammatory response at a 53-56 Mb interval in chromosome 2 [38] suggesting that the selection process of AIRmax and AIRmin lines might have affected the HCC susceptibility QTL mapped at chromosome 2. The chromosome 9 QTL interval contains several genes involved in inflammation and/or in cancer such as Caspase 1 (*Casp1*) and pannexin 1 (*Panx1*), involved in the release of mature inflammatory IL-1*β* [39]. In ABF4 mice, possible candidate gene mapping in the chromosome 7 QTL is *Fancf* (Fanconi anemia complement group F). Complications of Fanconi anemia include the development of liver tumors and other cancers [40]; *Fcgrt* gene (Fc receptor, IgG, alpha chain transporter) codes for hepatic FcRn regulate albumin homeostasis and susceptibility to liver injury enhancing sensitivity to albumin bound hepatotoxins [41]; *Fgf21* (fibroblast growth factor 21) is a novel biomarker for nonalcoholic fatty liver disease (NAFLD) in humans and limits ethanol-associated hepatotoxicity in mice [42]; and *Saa1* (serum amyloid A1) is an evolutionary highly conserved proinflammatory acute phase protein. Saa is predominantly secreted by hepatocytes and modulates fibrinogenesis by inducing inflammation, proliferation, and cell death in hepatic stellate cells [43].

We observed also coincidences with other QTLs such as *Lfibq14* and *21* for liver fibrosis involved in the regulatory network that determines the vulnerability to hepatic chronic injury and *Ap7q*, for alcohol preference [44, 45].

We determined the liver gene expression profile of AIRmax and AIRmin mice as an additional tool to identify candidate genes, because the transcriptome of normal tissue has been associated with genetic predisposition to tumorigenesis in different organs and experimental models. We observed that several transcripts with potential contribution to HCC susceptibility differ significantly in the normal liver from the two mouse lines. For example, genes grouped into functional categories such as defense response (lymphocyte antigens, major histocompatibility complex genes, integrins, and defensin) and cell cycle (cyclin) and *serpina1d* are upregulated in AIRmin livers. *β*-Defensin-1 behaves as a tumor suppressor protein in some human tumor types [46]. The vascular noninflammatory molecules Vanin pantetheinases (Vnn) are potential therapeutic targets in inflammatory diseases; Vanin genes influence the lipid profile and studies report a role for Vanin in inflammation, oxidative stress, cell migration, and numerous diseases [47]. H2Ea: mice bearing the H2b haplotype do not express I-E MHC class II molecules, due to deletion of the promoter region of the Ea gene. We verified before that the majority of AIRmax mice bear the H2b haplotype which might underlie the absence of H2Ea expression in the liver as well as in all tissues tested so far [11, 12]. AIRmax mice are susceptible to autoimmune diseases such as pristane-induced arthritis, and the E-alpha chain was described as protective to the development of systemic lupus erythematosus [48].

On the other hand, several CYP450 genes, implicated in xenobiotic metabolism, and genes involved in lipid metabolism are upregulated in AIRmax livers. The sphyngosine-1-receptor 5 (*Sp1r5*) gene maps at 21 Mb in chromosome 9, inside the QTL confidence interval. The bioactive lipid mediator sphingosine-1-phosphate (S1P), through activation of cell surface receptors S1PR1 to S1PR5, is involved in multiple cellular signaling systems and has a pivotal role in immune cell functions, inflammation, and cancer due to the control of immune cell trafficking. NK cells abundantly express S1PR5 and S1PR5 responses play a role in the trafficking of these cells from the bone marrow and lymph nodes into the tissue [49, 50]. It was previously demonstrated that AIRmax mice had higher relative numbers of NK cells in the spleen, as well as higher cytolytic activity against Yac.1 target cells than AIRmin mice [51]. NK cells are involved in the clearance of infectious agents and antitumor surveillance [52]; however, these cells can accelerate liver injury by producing proinflammatory cytokines and killing hepatocytes [53]. The S1P axis has been implicated in cancer and inflammatory diseases, and *S1pr5* is a good candidate modulator of the differential inflammation and HCC susceptibility of AIRmax and AIRmin mice [54].

In conclusion, our results show that the divergent susceptibility of AIRmax and AIRmin mice to chemically induced liver tumors correlates with specific hepatic gene expression profiles in untreated mice and the early production of inflammatory mediators in the liver following carcinogen injection. Linkage analysis in F2 (AIRmax×AIRmin) mice, as well as in ABF4 populations, revealed QTLs for HCC, in chromosomes 2 and 9, and 7, respectively. Genes and QTLs for phenotypes relevant for liver cancer, such as inflammatory response, liver fibrosis, alcoholism, obesity and lipid metabolism, and Fanconi anemia, also map to these regions. The experiments evidenced genetic heterogeneity in the control of predisposition to liver cancer and the role of inflammatory mediators secreted by resident or infiltrating cells in tumor development.

## Figures and Tables

**Table 1:** Parameters of liver tumor development in AIRmax, AIRmin, F1 (AIRmax×AIRmin), and F2 (AIRmax×AIRmin) intercross mice at 34 weeks after urethane treatment.

	Liver tumors		
Mice (*n*)	Mean number (*N*) (incidence %)	N_2^∗^	Mean volume (mm^3^)
AIRmax ơ (11)	12.5 ± 5.2 (100%)	7.3 ± 3.5	142 ± 69
AIRmin ơ (9)	0	0	0
F1 ơ (15)	0.4 ± 0.2 (27%)	0.3 ± 0.1	2 ± 1
F2 ơ (335)	1.4 ± 0.3 (51%)	0.8 ± 0.1	30 ± 6

^∗^N_2: number of liver tumors with diameter > 2 mm. Values expressed as arithmetic means and standard errors.

## Data Availability

The data used to support the findings of this study are available from the corresponding author upon request.
